# Infrastructure for Personalized Medicine at Partners HealthCare

**DOI:** 10.3390/jpm6010013

**Published:** 2016-02-27

**Authors:** Scott T. Weiss, Meini Sumbada Shin

**Affiliations:** 1 Partners Personalized Medicine, Partners HealthCare System, Brigham and Women’s Hospital, Harvard Medical School, Boston, MA 02115, USA; 2 Partners Personalized Medicine, Partners HealthCare System, Boston, MA 02139, USA; msumbadashin@partners.org

**Keywords:** personalized medicine, academic medical centers, Partners HealthCare, biobank, bioinformatics, laboratory testing, information technology infrastructure

## Abstract

Partners HealthCare Personalized Medicine (PPM) is a center within the Partners HealthCare system (founded by Massachusetts General Hospital and Brigham and Women’s Hospital) whose mission is to utilize genetics and genomics to improve the care of patients in a cost effective manner. PPM consists of five interconnected components: (1) Laboratory for Molecular Medicine (LMM), a CLIA laboratory performing genetic testing for patients world-wide; (2) Translational Genomics Core (TGC), a core laboratory providing genomic platforms for Partners investigators; (3) Partners Biobank, a biobank of samples (DNA, plasma and serum) for 50,000 Consented Partners patients; (4) Biobank Portal, an IT infrastructure and viewer to bring together genotypes, samples, phenotypes (validated diagnoses, radiology, and clinical chemistry) from the electronic medical record to Partners investigators. These components are united by (5) a common IT system that brings researchers, clinicians, and patients together for optimal research and patient care.

## 1. Introduction

In this article we will describe the resources devoted to Personalized Medicine at Partners HealthCare, how we integrate that set of resources with the existing infrastructure at the two academic medical centers: Massachusetts General Hospital and Brigham and Women’s Hospital and how we advance our mission to better integrate genomic data into clinical practice at Partners in a cost effective way. This article will describe the center and its components and relate each element to our overall vision and mission.

## 2. Results and Discussion

### 2.1. Partners HealthCare System

Partners HealthCare System (Partners) is a not-for-profit, integrated health care system in Boston, Massachusetts founded by two of the nation’s leading academic medical centers (AMC), Massachusetts General Hospital (MGH) and Brigham and Women’s Hospital (BWH), which have been ranked #1 and #6 respectively in the U.S. News and World Report 2015 Honor Roll of academic medical centers. In addition to the two AMCs, Partners also includes community and specialty hospitals, a physician network, community health centers, home care, and other health related services, cares for four million patients with more than 7000 physicians attending and has 160,000 admissions per year. The composition of the patients in terms of race, gender and age is representative of the population of eastern Massachusetts. In addition, Partners institutions maintain a total research budget of more than $1.4 billion. MGH and BWH are the largest private hospital recipients of National Institutes of Health (NIH) funding in the nation. 

### 2.2. Partners HealthCare Personalized Medicine (PPM)

The mission of the PPM is to utilize genetics and genomics to improve the care of patients through the promotion and implementation of personalized medicine in caring for patients throughout the Partners HealthCare System and in healthcare nationally and globally in a cost effective manner. Harvard Medical School (HMS) and Partners established the Harvard-Partners Center for Genetics and Genomics (HPCGG) in 2001, recently renamed as Partners Personalized Medicine (PPM) to reflect a heightened focus on translational issues related to moving genetics and genomics into clinical practice.

**Figure 1:**
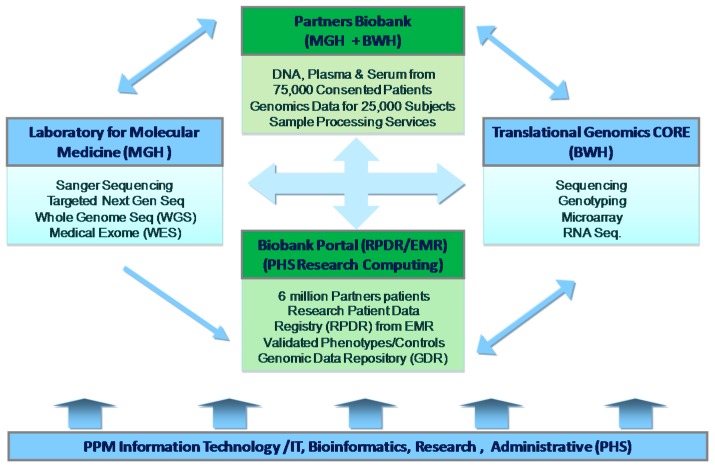
Partners HealthCare Personalized Medicine (PPM) organizational structure.

Figure 1 depicts the organizational structure of the Center. The arrows indicate informatics links between the various Center components: the Biobank, the Translational Genomics Core (TGC), the Laboratory for Molecular Medicine (LMM), and the Research Patient Data Registry (RPDR) to enable data analysis and distribution to investigators across the health system. One of the keys to PPM’s success is having a robust IT infrastructure that includes a sample management and tracking system (StarLIMS) and a genomic results delivery system GeneInsight (described below) and a home-grown LIMS System (GIGPAD). The details of this IT infrastructure are provided in subsequent sections. The Center is physically located at 65 Landsdowne St. in Cambridge, MA approximately 15 min from BWH and MGH. All staff are located onsite and each of the Labs (Biobank, LMM, and TGC) are contiguous to allow the use of the same equipment and resources. We will describe each of the components in Figure 1 in more detail.

#### 2.2.1. The Laboratory for Molecular Medicine (LMM)

The LMM is a CLIA-certified molecular diagnostic laboratory, operating within the PPM. The LMM was founded 13 years ago with the mission to bridge the gap between research and clinical medicine, by accelerating the adoption of new molecular tests into clinical care. The current focus of the LMM is on germ line mutation testing. Cancer testing is performed through molecular pathology laboratories at each AMC affiliated with the two cancer centers associated with Partners *i.e.*, the Dana Farber Cancer Center and MGH Cancer Center. Major areas of expertise include inherited respiratory disorders, cardiomyopathies, hearing loss, connective tissue disorders, RASopathies, and multi-organ genetic syndromes. Annually, the LMM performs about 5000 high complexity genetic and genomics tests of disease-targeted NGS panels for a variety of disorders with genetic and clinical heterogeneity, covering about 400 genes, as well as exome and genome sequencing. The lab consists of six geneticists and 25 staff including genetic counselors and fellows. The LMM continues to develop novel genetic tests in multiple areas, most recently, pulmonary and renal panels. An integral component of the mission of the LMM is the incorporation of IT support into the dayto-day operations of the clinical lab, as well as implementing innovative programs to help physicians stay current on genetic information relevant to their patients. The LMM shares the lab instruments with TGC and Biobank see Section 2.2.2 and Section 2.2.3 below. In addition, the LMM’s close integration with the other PPM components, especially with IT and bioinformatics teams, and its access to the AMCs physicians have allowed the LMM to have a proven track record of developing and clinically implementing novel, cutting-edge technologies, including bioinformatics tools, data analysis pipelines and novel approaches to interpret and communicate medical genomic results to healthcare providers and patients such as GeneInsight see Section 2.2.5 below. While many academic health centers have molecular genetic clinical laboratories, what is unique at PPM is the close link with the other two center’s labs (TGC and Biobank) and the common IT Infrastructure for all components [1].

#### 2.2.2. Translational Genomics Core (TGC)

The Translational Genomics core of the PPM (Figure 1) performs high throughput next-generation sequencing (NGS), library construction for NGS, genotyping, and gene expression analysis (both chip and sequencing) for all Partners investigators and non-Partners researchers. The core consists of one Director, one Lab Manager, one Project Manager, and four Technicians. The core serves over 200 customers per year, performs over 400 individual projects annually, and supports over $148 million in NIH Grants. The core has one Illumina HiSeq 2500 and two Illumina MiSeq for sequencing. It performs sequencing of large genomes and transcriptomes, whole exome, and whole genome for both clinical (LMM) and research projects. The core provides flexible, high-throughput SNP genotyping using the Illumina iScan platform. Both Illumina (HT-12) and Affymetrix chip based microarray assays are supported as well as RNA seq. The core is currently developing end to end services for sequencing of the human microbiome as well as RNA seq for microRNA in tissue, serum and plasma. Genome Wide SNP genotyping with the Illumina Mega Chip (GWAS) is being performed on the first 25,000 subjects in the Biobank. The first 5000 subjects have been available in the Fall of 2015 with the full 25,000 available in 2017 see (Figure 3 below) [2].

#### 2.2.3. Partners HealthCare Biobank

Partners HealthCare Biobank is a large research data and sample repository operating within the framework of PPM (Figure 1). It provides researchers access to high quality, consented samples to help foster research, advance our understanding of the causes of common diseases, and advance the practice of medicine. The Partners Biobank provides banked samples (plasma, serum and DNA) collected from consented patients. These samples are available for distribution to Partners Healthcare investigators with appropriate approval from the Partners Institutional Review board (IRB). They are linked to phenotypic data stored in the Research Data Patient Registry (RPDR), as well as some additional health information collected at the time of collection. To date, more than 35,000 patients have consented to join the Partners Biobank. An additional 1000 to 2000 patients consent each month. The ultimate goal is to reach 75,000 total subjects of whom samples are available on 50,000. Samples are collected at the participating hospitals within Partners HealthCare. Samples are sent to the processing labs where the plasma, serum, and buffy coats from each specimen are isolated, with the intention of being completed within 4 h of collection to ensure highest quality for banking purposes. All specimens are shipped to the Central Facility for DNA Extraction and long-term storage. One of the buffy coat aliquots is extracted for DNA, quantitated and stored in both master tubes and tubes containing 50 μg of DNA. These specimen shipments are tracked by our internal software LIMS (STARLIMS), that interfaces with other software maintaining specimen and data integrity. STARLIMS manages the collection, processing, storage, distribution and billing of samples at the Central Facility. Samples are distributed in the following manner. As noted above, investigators may request assistance from Biobank co-Principal Investigators or complete specimen requests in the Biobank Portal of the Research Patient Data Portal. These requests are routed through our custom software (EMSI) for parsing, and then forwarded to the appropriate bank supervisor(s) for fulfillment. The Biobank Program Director is responsible for managing the day-to-day operations including the distribution of the samples to the investigator community, and ensuring that sample status is updated in Sunquest or Crimson the software used to track specimens, as appropriate. To date, the Biobank has distributed >5000 specimens to >50 different investigators through the RPDR and outreach to different clinics within the cardiovascular disease and various others. The Biobanks supports over $80 million in NIH research. The Biobank staff includes five Faculty members, one Program Director, one Project Manager, two Recruitment Managers, 18 Research Assistants, one Senior Lab Manager, and eight full time Technicians. As noted above, the central facility is co-localized with the Translational Genomics Core (TGC), allowing for tight integration and continuity of projects for investigators. Samples can be moved directly from the Biobank to TGC for genotyping/sequencing before results are returned to investigators. The biobank can accommodate >2 M specimens in 28.8 cubic-foot Revco Upright Ultra-low temperature freezers. To ensure specimen integrity, freezers are all on emergency back-up power. Each individual freezer is also monitored 24 h/7 days per week using the SIEMEN’s security system. This system is triggered when there is a loss of normal power, a rise in temperature within the freezer, or a loss of communication with the freezer’s alarm circuit. The storage space has two sources of cooling so that the back-up system (the independent HVAC) can function automatically upon loss of normal power through the use of an automatic transfer switch connected to the house standby generator. A copy of the Biobank consent has been included as a supplement to this article [3].

#### 2.2.4. PPM IT Infrastructure and Bioinformatics Team

PPM provides an integrated IT architecture supporting research and clinical activities, which is directly connected to the Partners and Harvard Medical School networks and to the rest of the academic community through the Internet. The PPM clinical IT team is responsible for IT support for LMM and GeneInsight [4]. The PPM Research IT is responsible for IT support for Biobank and TGC [4,5]. The PPM IT teams are part of Partners Research Computing group, which maintains PPM’s high performance computing infrastructure database servers and virtual machine servers that are currently used by our group in many applications. This includes ~100 Tb of dedicated primary tier storage with access to >100 Tb of additional storage, as well as access to >300 Tb of long-term replicated storage. We also have three dedicated computational clusters consisting of ten 128-core nodes with 128 GB of memory each, fourteen 72-core nodes with 96 GB of memory each, and sixteen 32-core nodes with 16 GB of memory each. All systems are patched, monitored and scanned routinely for vulnerabilities and intrusions by the systems administrator and Partners Information Security. In addition to our IT hardware, PPM has a dedicated full-time IT staff of 21, including directors, architects, analysts, developers, an implementation manager, implementation engineers, a quality manager, genetic counselors and a geneticist.

Three pieces of software tie the LMM, TGC, and Biobank together: GeneInsight (described below), GIGPAD, and StarLIMS. GIGPAD is the internal LIMS for LMM and TGC and handles samples management for these two labs. StarLIMS handles sample management for the Biobank and can hand these samples off to GIGPAD either in the LMM or in the TGC. StarLIMS is a commercial biobank software while GIGPAD and GeneInsight are home-grown.

The bioinformatics team consists of one director, and seven bioinformaticians. The bioinformatics team routinely processes and analyzes DNA sequencing, RNA sequencing, and microarray data, supporting both our CLIA-certified lab and outside investigators via the translational genomics core. This includes a custom automated pipeline for generating sample-specific, demultiplexed fastq files for all Illumina sequencing projects. For DNA-sequencing: (1) Quality Control steps are performed using Picard and SAMtools; (2) alignment and variant calling use BWA and GATK for indels and small SNVs; (3) coverage metrics are generated with a combination of GATK and custom scripts; and (4) CNV calls are generated with a custom tool VisCap. Additionally, an evaluation of CNV calling using XHMM, ExomeDepth, and others is underway. Our RNA-seq pipeline uses (1) FastQC for quality steps and (2) the Tuxedo package for differential expression analysis and visualization, including TopHat, Cufflinks, CuffDiff and CummeRbund. Microarray data is processed via (1) Affymetrix Expression Console; (2) Illumina GenomeStudio; and (3) Heatmap2 package in R and custom scripts for visualization. We are also in the process of implementing Beelin/Autoconvert, plink, and custom software for high-throughput genotyping and annotation of Illumina data.

#### 2.2.5. GeneInsight Suite

**Figure 2:**
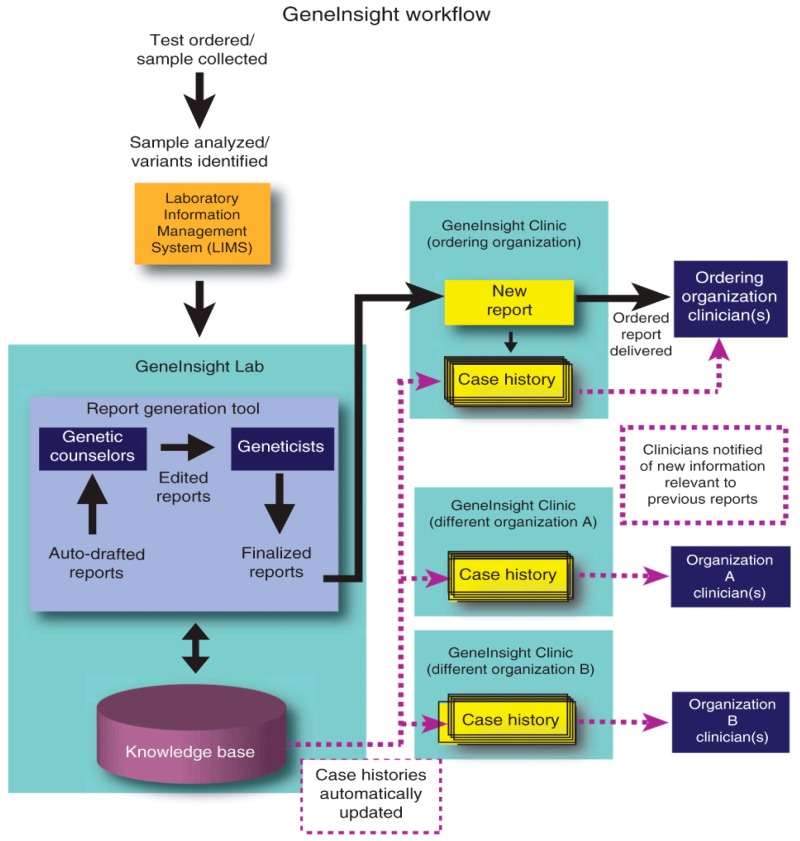
GeneInsight workflow.

The GeneInsight Suite of IT tools has been developed by the PPM Clinical IT team, to address some of the most critical challenges to enabling broad clinical utilization of genomic testing, a key step towards the promise of personalized medicine. These challenges include the need to streamline the clinical testing process, manage the vast amounts of data generated through genetic testing, generate clinically useful interpretations from these data and channel this information efficiently and effectively to clinicians to impact patient care. GeneInsight^®^ assets have been developed through close collaboration between LMM laboratory technicians, laboratory managers, geneticists, IT developers and Partners hospitals practicing physicians to address the distinct, yet interrelated, needs of laboratories and providers. GeneInsight delivers the IT infrastructure needed to overcome these challenges (Figure 2).

IT applications include: GeneInsight Lab^®^ (Boston, MA, USA), a laboratory tool to assist with genetic variant knowledge management and interpretative report generation, GeneInsight Clinic^®^, a standalone hosted clinician interface to enable delivery of patient genetic test results and future variant updates to clinicians, and GeneInsight Network, a hub designed to enable high throughput transfer of structured genetic data between and among laboratories and clinicians. The system has been used to generate over 30,000 clinical reports. The GeneInsight Suite is registered with the FDA as a Class I exempt medical device. It is subject to inspection and must comply with quality regulations. Based on a set of IT assets designed to support genetic testing, the PPM IT team has built a solution to provide broad support for the genetic testing processes in clinical settings. At present a strategic alliance has been formed between Sunquest Information Systems and Partners HealthCare around GeneInsight to collaborate on providing seamless genetic testing workflow capabilities to clinical geneticists and pathologist and the goal to provide a wider dissemination of the software and its continued development into the industry standard for delivery of genomic results to clinicians [6].

### 2.3. Partners Research Computing

Partners Research Computing is a Division of Academic Programs of Partners HealthCare. The group occupies about 5000 square feet about 1200 yards from the MGH main campus and is physically separate from the PPM space at 65 Lansdowne Street, Cambridge. This space is connected through Ethernet with the main campus. The space houses offices for about 27 employees and staff members. Dr. Murphy’s computer resources are located in the Needham Data Center of Partners HealthCare. Over 40 powerful Windows 2003/2008 Pentium IV class servers are available on site, including several that host large Oracle and SQL Server databases. These servers host both relational database (Oracle and SQL Server) and Web server (Microsoft IIS and Tomcat/JBoss) software. Database servers include RAID5 disk array capabilities. There are also redundant Pentium IV class Windows 2003 file servers for shared use. In total the group hosts over 20 Terabytes of server disk storage. All servers are backed up nightly to a Tivoli Storage Manager (TSM) system. Development and production servers reside in the Partners corporate datacenter, which is, staffed 24/7. The main enterprise computer systems at Partners are available through the network. All network activity at Partners occurs behind a Cisco Firewall and traffic is constantly monitored. The group has over 50 desktop workstations ranging from high-end Pentium multi-processor systems to moderate Pentiums. They include machines with Microsoft Windows, Macintosh OS, and Linux operation systems. This group has built the Research Patient Data Registry including the Biobank portal and the Phenotype Discovery Center as described below.

#### 2.3.1. Research Patient Data Registry (RPDR) and Biobank Portal

Developed by the Partners Research Computing group, the RPDR is a data warehouse that gathers data from multiple hospital electronic record systems at Partners HealthCare and stores it in a SQL Server database. The RPDR gathers clinical data from several hospital systems at Partners HealthCare. (Enterprise Master Patient Index—EMPI, Hospital Decision Support System—EPSI (formerly TSI), Physician Billing System—IDX and EPIC, Longitudinal Medical Record—LMR, Corporate Provider Master—CPM, Clinical Data Repository—CDR, and Partners Personalized Medicine—PPM), and stores the data in one central data warehouse. Researchers are able to query this data by using an online query tool.

**Figure 3:**
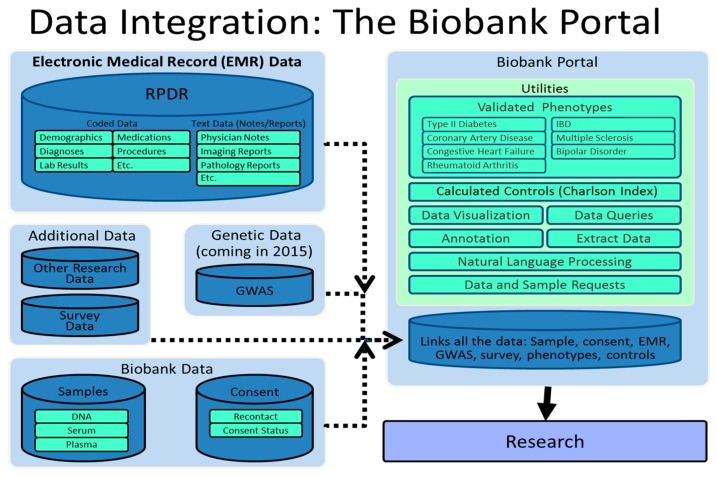
Data Integration—Biobank Portal.

The query tool returns aggregate totals of patient data that are populated with appropriately obfuscated, de-identified/encrypted data as per HIPPA privacy rules and the HHS Common Rule. With the proper IRB approval, researchers may access the patients’ detailed medical records for their specified cohorts of patients. The detailed medical records are returned to researchers in an encrypted Microsoft Access file and text (.txt) files. Detailed medical records may include the following types of data: transfusion, cardiology, contact information, demographics, diagnoses, discharge notes, endoscopy, laboratory tests, PEAR allergies, LMR health maintenance, LMR medications, LMR notes, LMR problems, LMR vital signs, medications (RxNorm), microbiology, operative notes, pathology reports, procedures (CPT codes), providers, pulmonary, radiology reports, radiology tests and transfusion. Furthermore, images from hospital image repositories can be returned and viewed online. The RPDR is able to obtain patient notes from hospital systems and create a secure database for eMERGE III [7]. Security and privacy of the patients whose data are contained in RPDR are of paramount importance in its operation. We have robust methods to protect the information while maintaining its usability. We have developed methods of data obfuscation to allow users to have access to aggregate data without threatening patient confidentiality. In addition to the RPDR, both genotype data (GWAS) and survey data on Biobank participants are visible in the Biobank Portal that is the final common pathway for investigators to get all of this integrated data. Figure 3 depicts the infrastructure for the Biobank Portal [8].

#### 2.3.2. Phenotype Discovery Center (PDC)

The Partners Phenotype Discovery Center provides support to investigators to link phenotypes with data on consented subjects in the Partners HealthCare Biobank. As part of the PDC, we have created the Biobank Portal (see Figure 3) to combine specimen data with data from the electronic medical record in a SQL Server database with a web-based application that enables users to query, view and work with the data in a variety of ways. The Biobank Portal allows users to perform queries, visualize longitudinal data (e.g., medication prescriptions, diagnoses, lab results), perform PheWAS based on >1500 clinically grouped ICD9-CM codes, query phenotypes defined by *i2b2* algorithms, perform automated natural language processing (NLP), and request samples from cases and matched controls. Data in the Biobank Portal database includes narrative data from doctors’ notes and other hospital text reports (cardiology, pathology, radiology, operative, discharge summaries), as well as coded data such as demographics, diagnoses, procedures, vital signs, lab values and medications. In addition, patient reported data from the health information survey given to all Biobank subjects is included in the Biobank Portal database and contains data on body mass index, occupational exposure, sun exposure, physical activity, alcohol, smoking and sleep behaviors, family history of disease and reproductive history for women.

Users of the Biobank Portal application can run queries on the aforementioned data to find particular sets of patients and then view the query criteria in a timeline patient-by-patient to visualize when phenomena of interest have occurred. Users can also review sets of patients in a Viewer that allows them to look at each patient’s data to determine whether or not to request specimen data for that patient. Users can then use the application to request the set of patients selected using the Timeline and Viewer from the Biobank. Another set of functions available in the Biobank Portal is designed to help with phenotyping sets of patients. Existing validated phenotypes are available in the user interface for eight diseases (Rheumatoid Arthritis, Ulcerative Colitis, Crohn’s Disease, Multiple Sclerosis, Type2 Diabetes Mellitus, Coronary Heart Disease, Congestive Heart Failure, and Bipolar Disorder), with an additional set of 12 planned to be completed over the next year. In addition, we are creating a Validation Workbench to help users create their own phenotypes, which will require creating a Natural Language Processing pipeline to help extract required features from narrative data, creating further methods to help users annotate the data, providing basic statistical guidelines and educating researchers about the nature of this work. Ultimately, genotyped results for the biobank subjects will be added into the Biobank Portal data mart and made available for further investigation. The Biobank is fully integrated with the Laboratory for Molecular Medicine, and the Translational Genomics Core via the IT connections in the Biobank Portal (Figure 1). All of the work done within the Biobank Portal and the Phenotype Discovery Center has been vetted by the IRB and abides by strict security measures.

## 3. Conclusions

Although we do not provide a formal analysis of similar programs at other AMCs in this description of the PPM, it is clear that the comprehensive nature of the infrastructure integration is not common in AMCs. Investigators can utilize the PPM to obtain consented samples, have them genotyped or sequenced, and then develop relevant diagnostic and prognostic indicators using the structured IT system across the three labs. We propose this as one model that will facilitate progress in this complex arena. We also note that the approach that we have taken is dynamic. We are moving in the direction of having not just the LMM approved, but also the TGC core and the Biobank CLIA, thus further strengthening the link between research and clinical care.

## References

[1] Rehm H.L., Hynes E., Funke B. (2016). The changing landscape of molecular diagnostic testing: Implications for Academic Medical Centers. J. Pers. Med..

[2] Blau A., Brown A., Mahanta L., Amir S. (2016). The Translational Genomics Core at Partners Personalized Medicine: Facilitating the Transition of Research towards Personalized Medicine. J. Pers. Med..

[3] Karlson E., Boutin B., Hoffnagle A., Allen N. (2016). Building the Partners HealthCare Biobank at Partners Personalized Medicine: Informed consent, return of research results, recruitment lessons and operational considerations. J. Pers. Med..

[4] Boutin N., Holzbach A., Mahanta L., Aldama J., Cerretani X., Embree K., Leon I., Rathy N., Vickers M. (2016). The information technology infrastructure for the translational genomics core and the Partners Biobank at Partners Personalized Medicine. J. Pers. Med..

[5] Tsai E.A., Shakbatyan R., Evans J., Rossetti P., Graham C., Shamra H., Lin C.F., Lebo M. (2016). Bioinformatics Workflow for Clinical Whole Genome Sequencing at Partners HealthCare Personalized Medicine. J. Pers. Med..

[6] Aronson S., Mahanta L., Hien L., Clark E., Babb L., Oates M., Rehm H.L., Lebo M. (2016). Information technology support for clinical genetic testing within an Academic Medical Center. J. Pers. Med..

[7] Smoller J., Karlson E., Green R., Kathiresan S., MacArthur D.G., Talkowski M., Murphy S., Weiss S.T. (2016). An eMERGE Clinical Center at Partners Personalized Medicine. J. Pers. Med..

[8] Gainer V., Cagan A., Castro V., Duey S., Ghosh B., Goodson A., Goryachev S., Metta R., Wang T., Wattanasin N. (2016). Using i2b2 to enable researchers to work with the Partners Biobank data and samples in the Partners Biobank Portal at Partners Personalized Medicine. J. Pers. Med..

